# Consequences of Academic Failure in Medical School: The Student’s Perspective

**DOI:** 10.7759/cureus.87996

**Published:** 2025-07-15

**Authors:** Justin Cox, Sarika Grover, Sophie Kharileh, Katherine Haber, Nicola Savage, Jonathan Fuller

**Affiliations:** 1 Barts and The London School of Medicine and Dentistry, Queen Mary University of London, London, GBR; 2 Medicine and Surgery, George Eliot Hospital NHS Trust, Nuneaton, GBR; 3 Medicine, George Eliot Hospital NHS Trust, Nuneaton, GBR; 4 Medicine and Surgery, South Warwickshire University NHS Foundation Trust, Warwick, GBR; 5 Medicine and Surgery, Somerset NHS Foundation Trust, Yeovil, GBR; 6 Institute of Health Science Education, Queen Mary University of London, London, GBR

**Keywords:** academic failure, medical school education, medical student perspectives, summative assessment, training progression

## Abstract

Background

Academic failure is a common and often constructive part of medical education. For many students, encountering setbacks can serve as valuable learning experiences that foster resilience, self-awareness, and professional growth. Medical students, often high achievers, typically struggle with a fear of failure. This is usually driven by misconceptions of the consequences and of remediation. This, in turn, results in psychological distress, anxiety, and burnout. Despite its inevitability, perceptions of academic failure remain poorly understood and under-discussed. This study aims to evaluate medical students’ understanding of academic failure, perceived consequences, and suggestions for improving institutional transparency and support.

Methods

A cross-sectional study was conducted in October 2023 via convenience sampling, using a semi-structured questionnaire to assess the perceptions of United Kingdom-based clinical medical students on their perceptions of the consequences of academic failure and their recommendations for institutional change. Data were thematically analysed to identify a consensus, common patterns, noteworthy responses, and alignment with literature.

Results

A total of 30 students were included in the study, of whom 19 (63%) self-reported a good understanding of academic failure, while 11 (37%) indicated poor understanding, largely due to institutional ambiguity. Key academic concerns included deregistration (n=11, 37%) and exam retakes (n=7, 23%). Twenty-two (73%) students perceived little to no career impact. Personal consequences were more prominent, with 17 (57%) citing mental health decline and 16 (53%) noting loss of self-worth. Twenty (67%) students emphasized the need for procedural transparency regarding failure and remediation, with 11 (37%) calling for clearer information, and 15 (50%) suggesting cultural shifts away from perfectionism. Many recommended open discussions about failure (n=13, 43%) and tailored support for struggling students (n=11, 37%).

Conclusion

Medical students demonstrate varied understanding of academic failure, with personal consequences outweighing academic or professional concerns. Uncertainty about remediation procedures exacerbates fear and distress. Increased institutional transparency and efforts to reduce the stigma, without trivialising failure, and proactive, targeted support could modulate fear of failure and improve outcomes. Future research should broaden the student cohort and scope, for example, to include a wider range of medical students at multiple stages of education to further inform comprehensive support strategies in medical education.

## Introduction

Medicine is inherently reliant on clinical judgment and probabilistic reasoning, which are also closely associated with uncertainty and risk. Physicians frequently make decisions informed by evidence and professional intuition. Consequently, failure within medical practice and education is common, academically and clinically [1-3]. Setbacks are a common experience for medical students during their undergraduate training [1,4], and while failure can serve as a constructive component of the learning process, enhancing both academic ability and clinical acumen [2], it may also be potentially harmful psychologically and academically [5].

Medical students are often characterized as high achievers, a trait that contributes to heightened academic pressures [6]. The continual cycle of assessment (Objective Structured Clinical Examination (OSCE), written exams, clinical attachments, etc.) at both undergraduate and postgraduate levels, combined with peer scrutiny and a prevailing culture of perfectionism [7,8], creates an environment in which failure occurs more frequently than many students anticipate [9]. This environment has been associated with increased stress and a higher incidence of mental health problems when compared to non-medical peers [1,5,6]. For instance, a 2019 survey revealed that 40% of medical students reported illness due to stress at some point during the academic year [7], while a 2021 study found that 49% met the diagnostic criteria for burnout [6].

Fear of failure, an anxiety of underperformance, is a significant source of stress in medical education [10,11]. Although not inherently problematic, when disproportional, it can contribute to academic procrastination, heightened anxiety [10], a decline in mental health and self-esteem [11], and poorer academic outcomes [12,13].

A recurring theme in the literature on fear of failure is uncertainty around failure policy and remediation procedure. When medical students are unclear about the potential consequences of failure, this uncertainty often manifests as apprehension [2]. Transparency regarding academic failure within medical education is notably lacking [9,12]. Enhancing students’ understanding of the implications of failure is crucial, as it empowers them to prepare for, respond to, and potentially prevent academic setbacks [12]. In addition, fostering realistic expectations regarding the consequences of failure can promote adaptive levels of stress and anxiety that serve a protective function in the learning process [2,9].

Despite the importance of this issue, there is limited research within undergraduate medical education within the United Kingdom (UK) exploring medical students’ perceptions and understanding of academic failure, an evidence gap that must be addressed to develop targeted interventions [1,5]. Thus, this study aims to explore UK-based clinical medical students’ understanding of the academic, personal, and professional consequences of academic failure and to seek their recommendations for improving clarity and support around this issue.

This article was previously presented as an oral presentation at the 2024 Association for the Study of Medical Education (ASME) Annual Scholarship Meeting on July 10, 2024.

## Materials and methods

Study design

This was a cross-sectional study conducted in 2023. It utilised an online semi-structured self-report questionnaire. This is comparable to similar studies [6,9] and provides an effective method of reaching a broad base of students, allowing for the thematic analysis necessary for this type of research [11,14].

Study population and sample size

A convenience sampling of 30 responses was taken from current medical students at Queen Mary University of London, London, UK. Only clinical year students were included in this study to ensure that those participating had a breadth of medical school experience, which for the sampled population begins in the third year. At the point of data collection, all participants had experience of summative assessment, including written examinations and the OSCE. Pre-clinical students were excluded. Previous failure was not grounds for exclusion. The questionnaire was distributed to student year groups using a QR code and URL link via email, student union online newsletters, union society pages, and medical social media Whatsapp groups and social media pages/websites. There was no participation incentivisation.

Study measures and tools

An initial pilot survey was used to create a validated questionnaire using content validation. The survey was scrutinised via feedback from independent reviewers (Table 1), which was used to optimise the structure and content of the final questionnaire. Feedback was discussed with the research team to optimise content and wording, to ensure internal validity is addressed. The final survey questionnaire (see Appendices) collected the respondents' current year of study, with an 'other' option to include intercalating students or enforce the exclusion criteria. Age and previous degrees were not included as it was felt that the scope of this study does not include exploring in depth the causes of the perceptions explored at present. It then assessed participants’ self-perceived understanding of academic failure in medical school, their perceptions of its educational, personal, and professional consequences, and their recommendations for improving institutional support and transparency surrounding academic failure, all using open-ended questions.

**Table 1:** Feedback results of pilot survey.

Response serial number	Feedback responses
1	Avoid Likert scales where qualitative results are preferred.
2	Consider using fewer and very focused open questions.
3	Ensure wording of the questions are specific and direct to avoid ambiguous responses.
4	Reduce unnecessary questions that do not focus solely on the study aim.
5	Shorten questions to prevent convoluted and confused responses.
6	The question on understandings of the consequences is very vague. This needs to be clearer and more specific to the study aim.
7	Collect demographic data from the students, specifically year group and possibly age and if they have previous degrees to analyse the impact experience may have.
8	The study brief should be more concise. Participants are unlikely to read large paragraphs of instruction, and this may result in confusion when they provide responses.
9	Use QR codes vs links to increase response ease and therefore turnout.

Ethical considerations

The study was approved by the Research Ethics Committee of Queen Mary University of London (approval reference: QMERC22.314) and was in line with the standards of the institution, as aligned with the UK Research Integrity Office, and in accordance with the Declaration of Helsinki. Informed consent was obtained digitally within the survey from all participants prior to data collection. Data was stored securely within the institute's official cloud storage to ensure confidentiality was maintained.

Data analysis

Data for each question was categorised using an inductive approach and analysed based upon the following four elements: (i) the consensus, (ii) common themes or patterns in responses, (iii) surprising, noteworthy, or anomalous responses not in keeping with the majority factions of answers, and (iv) how literature relates to the findings.

## Results

A total of 30 students were included in the study; 21 (70%) participants were in the fourth year, six (20%) were in the fifth/final year, two (7%) were intercalating after the third year, and one (3%) was in the third year.

Understanding of failure

**Figure 1:**
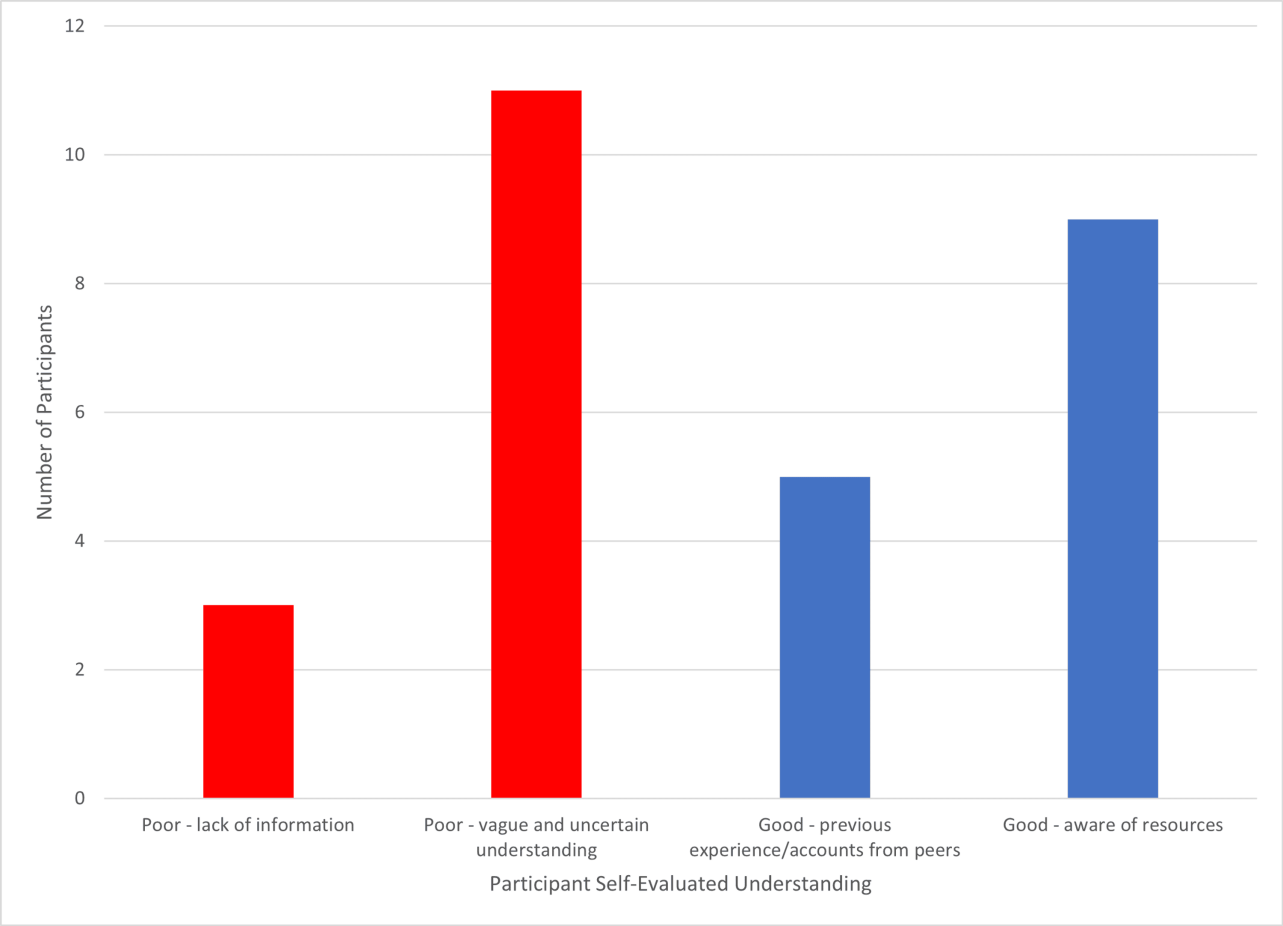
Participant reasoning for evaluation of their understanding of the consequences of failure.

When evaluating their understanding, 19 (63%) stated they had good knowledge, and 11 (37%) indicated poor knowledge, based upon inductive thematic analysis. All 11 who stated poor understanding cited uncertainty, and three (10%) highlighted a lack of information. Of the students who reported good understanding, nine (30%) were aware of the resources, such as handbooks and policies, available to them, and five (17%) had insight via previous experience either of themselves or their peers (Figure 1).

Consequences of failure

**Figure 2:**
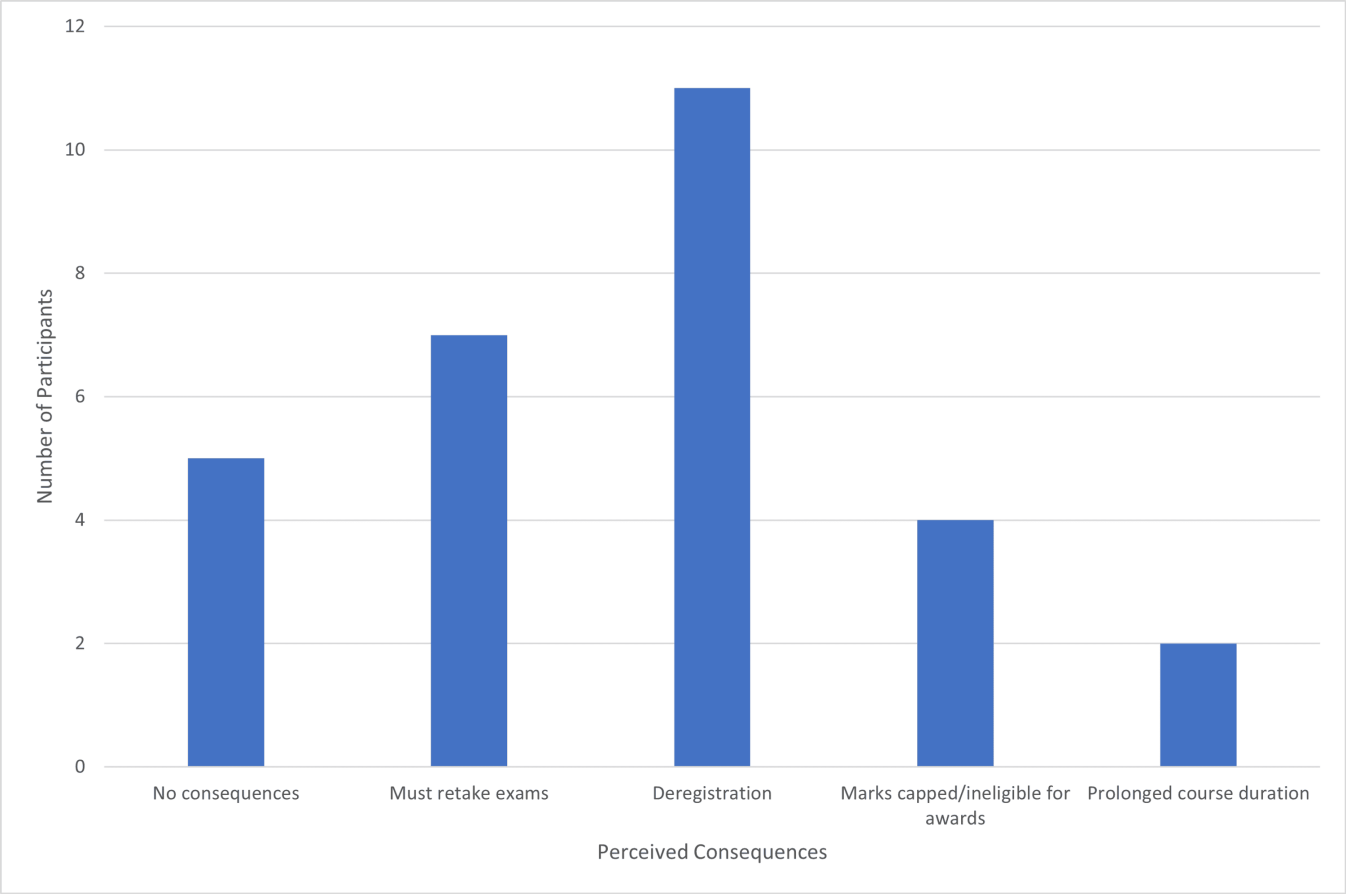
Participant perception of the consequences of failure during medical school.

When asked about perceived consequences during medical school, 11 (37%) students highlighted deregistration as a key concern. Seven (23%) mentioned re-taking exams, four (13%) stated capping of marks and being ineligible for awards, and two (7%) included prolongation of course duration. Five (17%) students reported there were no consequences (Figure 2).

**Figure 3:**
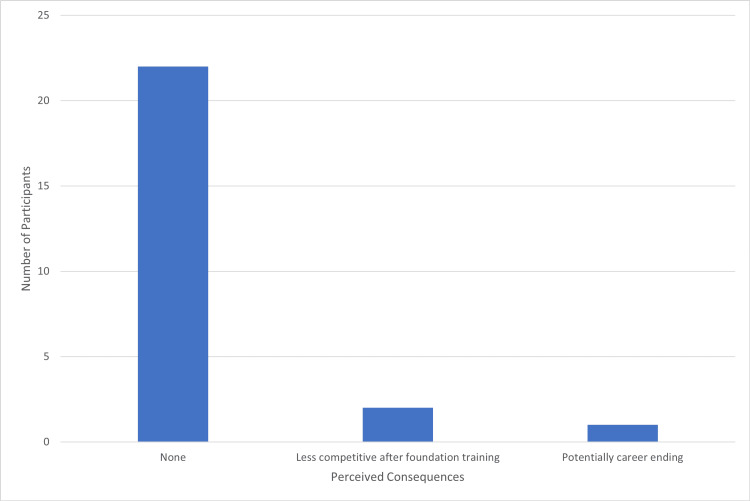
Participant perception of the consequences of failure for their career.

For career consequences, Figure 3 shows that 22 (73%) suggested there was no perceived impact post foundation training. Two (7%) thought they would be less competitive post foundation training, and one (3%) indicated it could potentially be career-ending.

**Figure 4:**
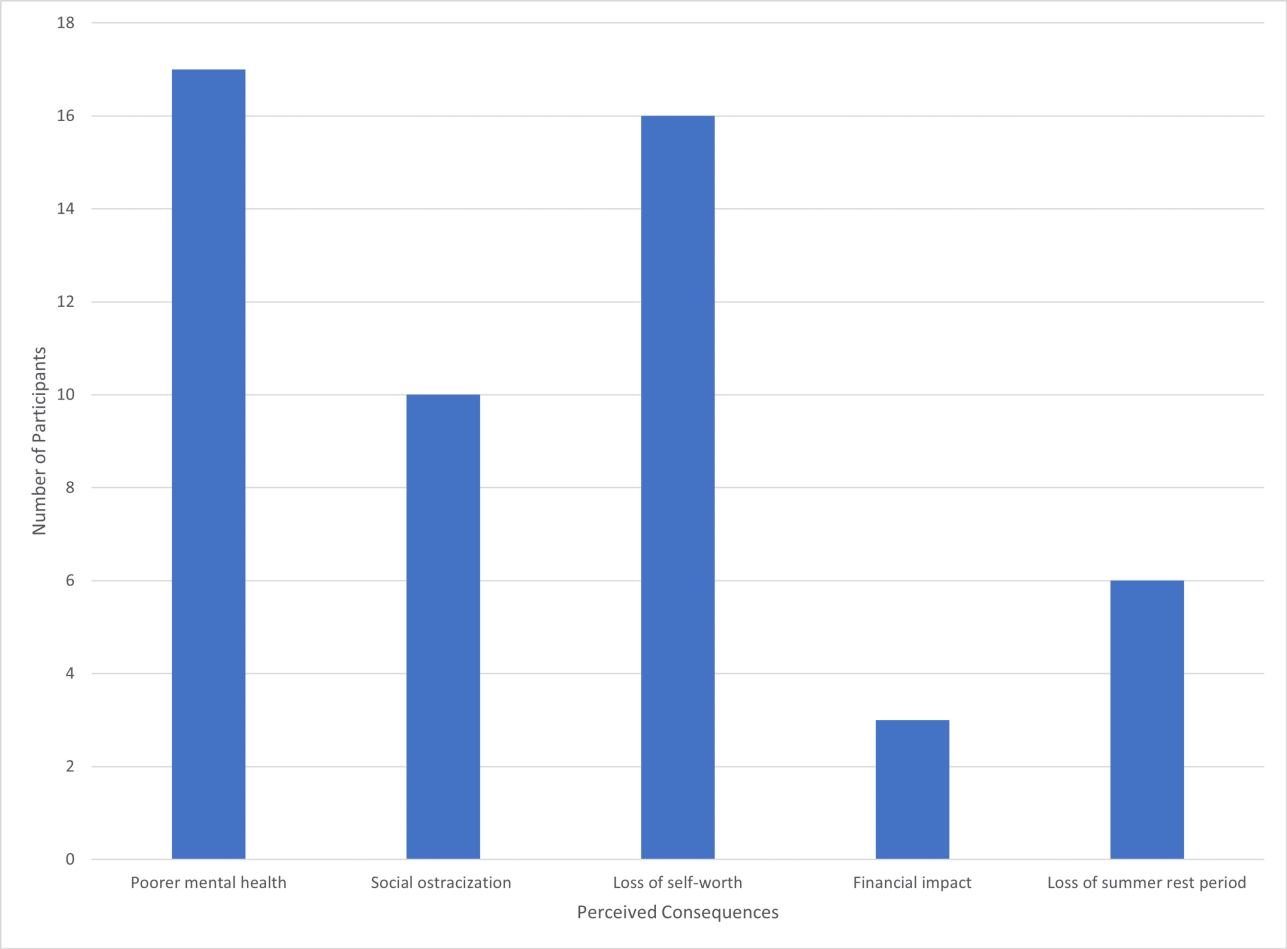
Participant perception of the personal consequences of failure.

Personal consequences reported were abundant. Seventeen (57%) students cited worsening mental health, 10 (33%) were concerned about social ostracization, and 16 (53%) feared loss of self-worth. Three (10%) included a financial burden, and six (20%) highlighted the loss of a summer rest period due to re-sitting exams (Figure 4).

Recommendations

**Figure 5:**
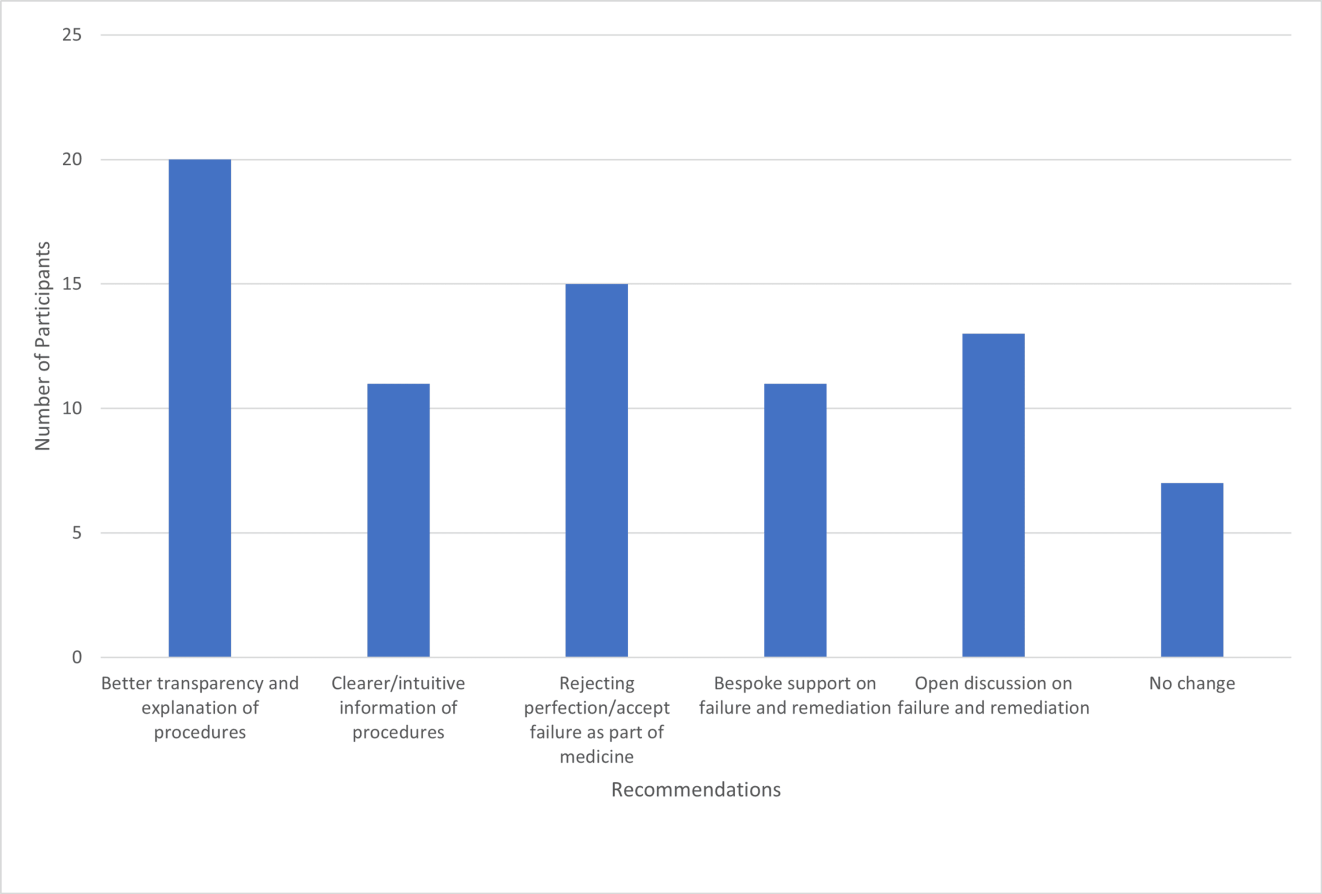
Participant recommendations for improving the approach to understanding failure in medical school.

When putting forward recommendations to improve understanding, 20 (67%) students called for better transparency and explanation of procedures, and 11 (37%) wanted clearer and more intuitive information. Fifteen (50%) participants called for a rejection of perfectionism in favour of acknowledging failure as an obstacle intrinsic to medicine, alongside 13 (43%) who suggested open discussion around failure and remediation. Eleven (37%) called for bespoke support around failure and remediation. Importantly, seven (23%) students stated that no change was warranted at present (Figure 5).

## Discussion

This study revealed significant variability in medical students’ understanding of academic failure, with perceived consequences largely centred on personal impact. While participant recommendations were diverse, several converging themes emerged, particularly regarding procedural transparency, the role of perfectionism, and the need for targeted support.

An important context for the population sampled is that students are informed formally of the processes of assessment procedures at the start of each academic year, alongside the process of progression and signposting to relevant policies and handbooks. Sources of support, both academically and pastorally, are also highlighted to students.

Understanding of failure

Students' understanding of academic failure ranged widely, linked with previous experiences and the knowledge, or lack of, how to seek appropriate information and policies. This suggests that while opportunities to develop insight do exist, current mechanisms for fostering this understanding are suboptimal. Students who demonstrated a more comprehensive understanding frequently cited personal or peer experiences, as well as familiarity with institutional policies and available support services. These factors align with prior research underscoring the value of shared experiences and procedural clarity in navigating academic challenges [10,15].

Conversely, poor understanding was commonly associated with uncertainty about institutional procedures and limited awareness of where to access guidance or remediation support issues, well-documented as exacerbating factors for stress and academic disengagement [3,7,8]. This highlights ongoing concerns about whether remediation processes are sufficiently transparent, accessible, and comprehensible to all students [2]. The heterogeneity in student responses supports the recommendation from previous studies, stating that support interventions should be actively targeted to more vulnerable students (identified by formative assessment, trends in results, or self-reporting concerns) rather than being applied universally [11].

Consequences of failure

Personal consequences emerged as the most prominent theme among respondents. Frequently cited outcomes included deterioration in mental health, social ostracization, and diminished self-worth. These issues mirror those reported consistently in the literature as key contributors to psychological distress and burnout among medical students [6,11,12]. This underscores the urgent need for interventions that proactively identify and support vulnerable students.

Concerns regarding academic consequences largely focused on remediation processes, with considerable anxiety around the potential for deregistration. These fears are reportedly amplified by the ambiguity of relevant institutional failure and remediation policies. Prior evidence suggests that clarity and consistency in procedural communication are essential for alleviating fear of failure and fostering successful remediation [6,14].

Interestingly, few participants expressed concern about long-term career impacts, suggesting that students tend to perceive failure as an acute, rather than an enduring, academic issue. Most believed that successful remediation would not result in a lasting mark, detrimental to their professional trajectory. Nonetheless, literature suggests that poorly managed remediation and failure experiences can have long-term consequences, reinforcing the importance of early and effective support to prevent recurrent academic difficulties [16].

Interestingly, one of the common recommendations made by students closely links to these personal consequences: the call for rejecting perfectionism, which may reduce a feeling of social ostracization. It is established that vulnerable students, or those who are experiencing significant fear of failure and a sense of social ostracism, are more likely to further isolate themselves and not seek help [4], even going as far as leading to extreme coping measures and avoidance [6,14]. Therefore, it is imperative that these students are particularly targeted for support and that these personal consequences are addressed as a priority.

Recommendations

A major theme among student recommendations was the need for greater transparency in remediation and failure procedures, alongside improved access to information about institutional policies. These suggestions are directly supported by studies linking poor understanding to procedural uncertainty and, conversely, improved understanding with previous experience or policy knowledge [1,8,9]. Enhancing transparency is a clear, evidence-based method for reducing fear of failure and improving outcomes.

Many students also expressed a desire for an institutional shift away from perfectionism, advocating for more open discussions around failure. Social ostracization was a prevalent concern as seen within the perceived personal consequences, and participants suggested that creating a constructive, non-judgmental culture could reduce stigma. Importantly, normalising discussion of failure without trivialising it can help students maintain a balanced perspective on academic challenges [17,18]. This cultural shift may also encourage the sharing of remediation strategies, which have been shown to improve understanding and reduce anxiety [7,10,15].

Targeted support and mentoring were also frequently recommended, which may take the form of staff tutoring or peer mentoring. Interventions aimed at vulnerable students, particularly those fearful of deregistration or uncertain about remediation pathways, have proven effective in other contexts [4,14], given that such students are less likely to seek help independently [6,14]. Proactive support must be sensitively delivered with attention to individual consent and preference. Notably, some students reported that they did not perceive a need for additional support, highlighting the importance of offering, rather than imposing, interventions.

Limitations

While the findings of this study are coherent with existing literature, several limitations must be acknowledged. The small sample size of approximately 2.2% of the population represents a constraint on both internal and external validity. Low response rates, common in online and self-reported survey research, were further influenced by limitations in study resources. Future research should aim to increase sample sizes and diversify recruitment strategies to enhance representativeness. Surveys may consider the inclusion of other question types, such as Likert scales, to provide additional dimensions of data and enhanced analysis.

Additionally, the use of self-report measures introduces the risk of recall and response bias. As in similar studies, perceptions may be influenced by the timing of data collection, particularly in proximity to summative assessments. Future studies could mitigate this by conducting longitudinal or periodic data collection and by refining the specificity and structure of survey instruments to enhance reliability.

## Conclusions

This study highlights the variability in medical students’ understanding of academic failure, and underscores the predominance of personal, rather than academic or professional, consequences. Uncertainty surrounding remediation procedures contributes significantly to fear and distress, particularly amongst vulnerable students. Clearer institutional communication and transparency in failure and remediation procedure, active support mechanisms, and a cultural shift away from perfectionism toward openness and normalisation of failure may mitigate these effects. Targeted, rather than universal, interventions are likely to be most effective.

Future research should further explore student perspectives across broader contexts such as multiple medical schools across multiple states and including students at all stages of education, to develop tailored educational and pastoral strategies within medical training on a national level.

## Figures and Tables

**Figure 6:**
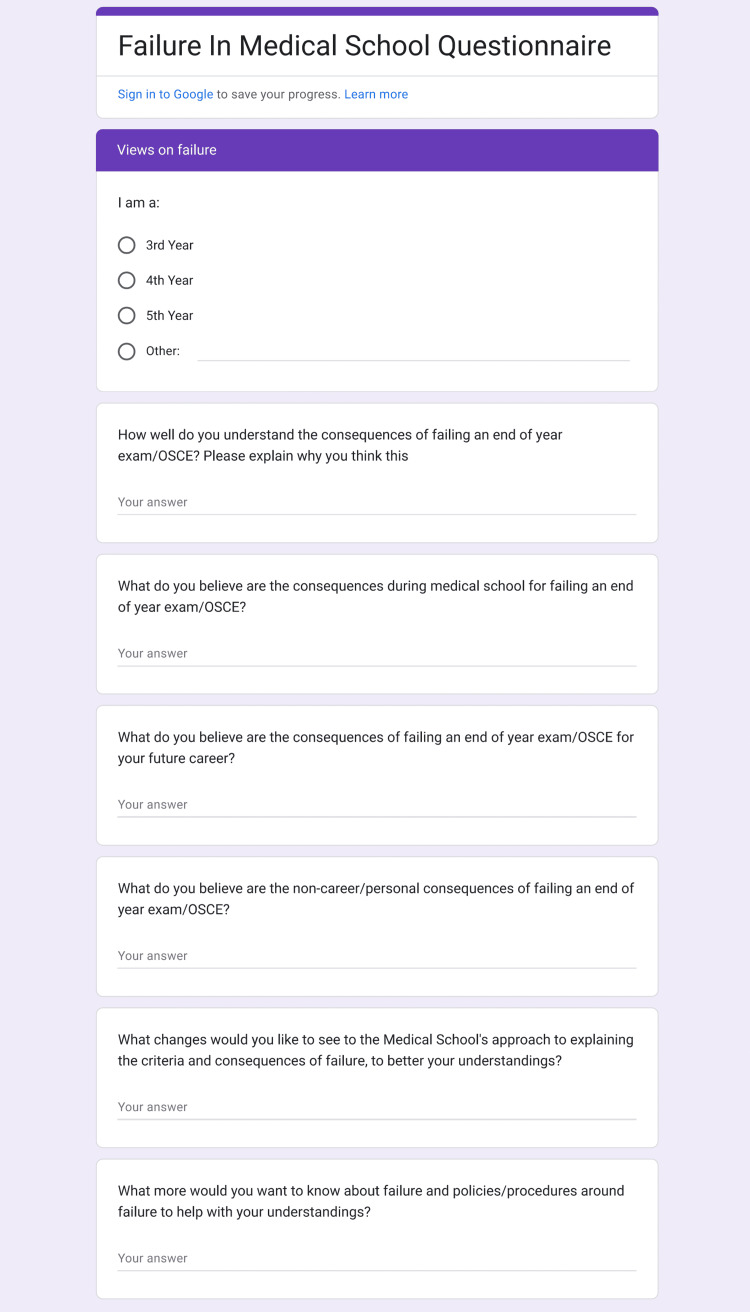
Study questionnaire
